# Development and Evaluation of a Competitive Enzyme-Linked Immunosorbent Assay Based on Swine Monoclonal Antibodies for Detecting Neutralizing Antibodies against Senecavirus A

**DOI:** 10.1128/spectrum.04599-22

**Published:** 2023-04-10

**Authors:** Xueqing Ma, Jiaxin Huang, Kun Li, Kailu Ding, Yuanfang Fu, Jing Zhang, Zhixun Zhao, Pinghua Li, Xingwen Bai, Dong Li, Xia Liu, Qiaoying Zeng, Zaixin Liu, Pu Sun, Zengjun Lu

**Affiliations:** a State Key Laboratory of Veterinary Etiological Biology, College of Veterinary Medicine, Lanzhou University, Lanzhou Veterinary Research Institute, Chinese Academy of Agricultural Sciences, Lanzhou, China; b National Foot and Mouth Disease Reference Laboratory, Lanzhou Veterinary Research Institute, Chinese Academy of Agricultural Sciences, Lanzhou, Gansu, China; c College of Veterinary Medicine, Gansu Agricultural University, Lanzhou, China; University of Prince Edward Island

**Keywords:** senecavirus A, monoclonal antibody, competitive ELISA, antibody detection

## Abstract

Senecavirus A (SVA) is an emerging viral pathogen related to vesicular disease and neonatal mortality in swine, which results in enormous economic losses to the global swine industry. The clinical signs of SVA are indistinguishable from those of other vesicular diseases, such as foot-and-mouth disease, which is an economically devastating animal disease. Therefore, development of a rapid, sensitive, and specific diagnostic method for the detection of SVA infection is critical for the prevention and control of SVA and would help to rule out other exotic diseases. In this study, two whole-porcine anti-SVA antibodies (1M5 and 1M25) were produced using single B cell antibody technology. 1M5 and 1M25 possessed neutralizing activity against SVA but recognized different conformational epitopes that depended on the intact virion. Using 1M5 as the capture antibody and biotinylated 1M25 as the detection antibody, a reliable and rapid competitive enzyme-linked immunosorbent assay for detecting neutralizing antibodies (NAC-ELISA) against SVA was developed. Receiver-operating characteristic curve analysis showed that the sensitivity and specificity of the assay were 98.11% and 100%, respectively, with a cutoff percent inhibition value of 45%. The NAC-ELISA was specific for detecting SVA-specific antibodies, without cross-reactivity to other virus-infected sera. The results of the NAC-ELISA showed a strong agreement with the results of the virus neutralization test. Therefore, the NAC-ELISA developed in this study represents a sensitive, specific, and reliable tool for the detection of SVA-specific antibodies, which is applicable for serodiagnosis and serological surveillance of SVA and is conducive to the prevention and control of SVA.

**IMPORTANCE** Senecavirus A (SVA) is an emerging picornavirus related to vesicular disease and neonatal mortality in swine, which results in enormous economic losses worldwide. Additionally, the clinical characteristics of the disease are indistinguishable from those of other vesicular diseases, such as foot-and-mouth disease. Therefore, developing tools for rapidly and accurately detecting SVA infection is critical and urgent. In this study, two porcine-derived monoclonal antibodies against SVA were generated, and a competitive ELISA for the detection of neutralizing antibodies (NAC-ELISA) against SVA was successfully developed using these two porcine monoclonal antibodies. The NAC-ELISA was SVA specific with no cross-reactivity to other related pathogens and had high sensitivity, specificity, and reproducibility for detecting SVA-specific antibody. Therefore, the NAC-ELISA developed in this study may be of great value as a simple and reliable tool for serodiagnosis or surveillance of SVA and may facilitate the prevention and control of SVA.

## INTRODUCTION

Senecavirus A (SVA), previously known as Seneca Valley virus, is a nonenveloped, single-stranded positive-sense RNA virus belonging to the genus *Senecavirus* of family *Picornaviridae* (1, 2). The SVA virion is an icosahedral symmetry structure with a diameter of approximately 25 to 30 nm (2, 3). The viral genome is approximately 7.2 kb in length and consists of a 5′ untranslated region (5′-UTR), a single large open reading frame (ORF), and a 3′-UTR. The ORF encodes a single polyprotein which is processed by virus-encoded proteases to generate four structural proteins (VP1 to VP4) and eight nonstructural proteins (L_pro_, 2A, 2B, 2C, 3A, 3B, 3C_pro_, and 3D) (1, 4, 5). The structural proteins are necessary for formation of mature viral particle, and 60 copies each of four structural proteins (VP1 to VP4) compose the viral icosahedral capsid. The nonstructural proteins are involved in viral RNA replication (6).

SVA was initially discovered and isolated from a human embryonic retinal cell (PER.C6) culture in 2002 and considered a contaminant in the cell culture (4, 7). After that, SVA was developed as an oncolytic agent in human cancer therapy but not associated with sporadic outbreaks of vesicular disease in pigs (8–10) until 2007 when SVA was first identified in pigs with idiopathic vesicular disease (IVD) in Canada (11). Subsequently, another case of SVA infection was identified in the United States in 2010. Since 2014, outbreaks of SVA-caused swine vesicular diseases (SVDs) have been reported in many countries, including Brazil (12–14), the United States (15), China (16–19), Thailand (20), Colombia (21), and Vietnam (22), indicating that SVA might circulate globally. Clinically, SVA infection mainly causes porcine vesicular lesions on the snout, coronary bands, oral mucosa, and hooves of pigs of different ages and acute death of neonatal piglets (23, 24). Thus, the spread and prevalence of SVA in pig herds could cause tremendous economic losses for the global porcine industry. Moreover, the symptoms of SVA infection are clinically indistinguishable from those of other vesicular diseases, such as foot-and-mouth disease (FMD), vesicular stomatitis (VS), vesicular exanthema of swine (VES), and swine vesicular disease (SVD) (23, 25). Since FMD is a highly contagious and devastating animal disease (26), any outbreak of vesicular disease in pigs should be diagnosed to exclude the possibility of an FMD virus (FMDV) infection. Hence, development of a rapid, sensitive, and specific diagnostic method for the detection of SVA infection is helpful to rule out infection by foreign animal vesicular disease pathogens and to prevent and control the spread of SVA.

At present, the common diagnosis methods for SVA mainly include reverse transcription-PCR (RT-PCR), virus neutralization test (VNT), indirect fluorescent antibody test (IFA), indirect enzyme-linked immunosorbent assay (ELISA), and competitive ELISA (cELISA) (13, 27–31). RT-PCR is a rapid, sensitive, and specific method to determine whether animals are acutely infected with SVA or whether vesicles contain SVA, but a negative result cannot rule out previous herd exposure since clinical symptoms of infection usually disappears within 1 to 2 weeks (13, 23). The presence of SVA-specific antibodies in serum may indicate previous SVA infection and the possible presence of SVA in a herd. IFA and VNT can be used to test for the presence of serum antibodies, and VNT is considered the gold standard for detection of neutralizing antibody in animal sera. However, IFA and VNT are too complex and time-consuming to be suitable for clinical field testing. In contrast, ELISA methods are simple, rapid, and easy to perform. The ELISA formats that have been extensively developed for detection of SVA antibodies include indirect ELISA and cELISA (30–33). These ELISAs are based on polyclonal antisera derived from rabbit or mouse monoclonal antibodies (MAbs) which were prepared from mouse hybridomas. However, rabbit polyclonal antisera and murine MAbs may have responses to SVA different in antigenic structure and preference from those of the natural host, resulting in false-positive reactions. Additionally, preparation of rabbit polyclonal antisera and murine MAbs requires animal immunization and is time-consuming and laborious. Furthermore, MAbs tend to be lost due to the instability of hybridomas. Nowadays, however, with the advent of single B cell antibody technology, many of these problems have been overcome. This technique is a combination of single cell sorting and single cell reverse transcription-PCR (34, 35), which allows for the isolation of single B cells with different antigen specificities by flow cytometry and the amplification of the cognate immunoglobulin light- and heavy-chain variable region (V_L_ and V_H_, respectively) genes from single B cells. These genes were then cloned and expressed in mammalian cells such as HEK293T or CHO-S cells (36–38). Given the characteristics of these methods, using the single B cell antibody technique to isolate functional MAbs has many advantages. First, this technique is species independent, and the MAbs obtained by this technique can maintain the original V_H_ and V_L_ pairing that exists in animals. Second, rare epitope-targeting antibodies can be obtained from rare, highly discrete B cell subpopulations. However, this technique is limited to B cells expressing a B cell receptor on the cell surface and by the availability of good antigenic baiting reagents. Several MAbs against HIV, classical swine fever virus (CSFV), FMDV, porcine reproductive and respiratory syndrome virus (PRRSV), and Zika virus have been obtained by this technology (39–43). However, the production of whole-porcine MAbs against SVA has not been reported.

In this study, two SVA-specific porcine MAbs were produced by the single B cell antibody technique, and a competitive ELISA for the detection of neutralizing antibodies (NAC-ELISA) against SVA was developed using these two porcine MAbs. The NAC-ELISA used porcine MAb 1M5 as the capture antibody and the other biotinylated porcine MAb, 1M25, as the detection antibody. Both 1M5 and 1M25 were neutralizing antibodies against SVA and derived from the natural host, the pig. The use of natural host porcine-derived MAbs is expected to improve the specificity and accuracy of ELISA in detecting neutralizing antibodies from natural hosts. The assay was validated using a panel of serum samples with known status. The results of the NAC-ELISA were comparable to those obtained by VNT. The NAC-ELISA developed in this study could be useful for serodiagnosis and serological surveillance of SVA and contribute to the prevention and control of SVA.

## RESULTS

### Production of MAbs 1M5 and 1M25.

**FIG 1:**
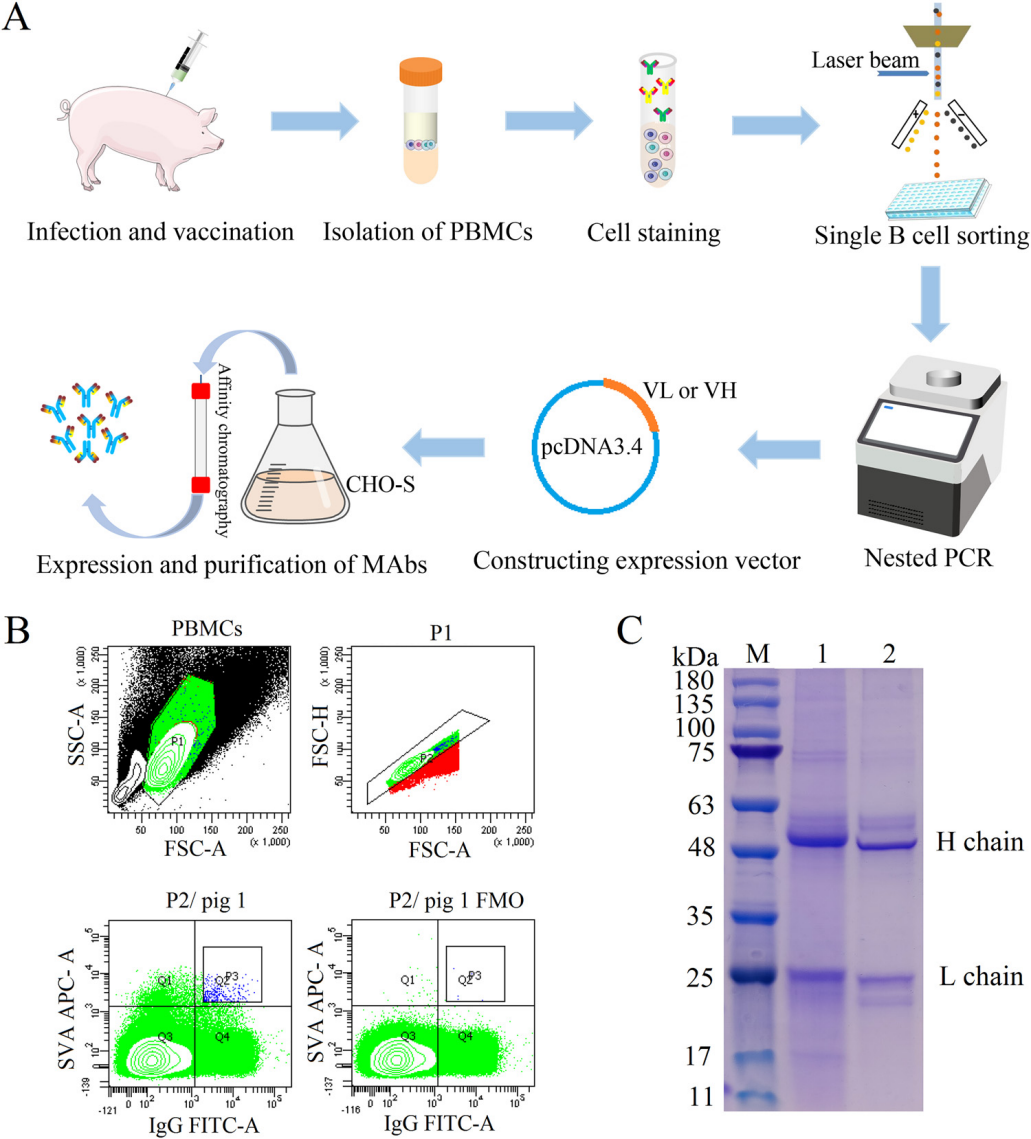
Production of the MAbs using singe B cell antibody techniques. (A) Pattern diagram of porcine MAb production using singe B cell antibody techniques. (B) Detection and isolation of SVA-specific IgG^+^ B cells by flow cytometry. FSC, forward scatter; SSC, side scatter. (C) Expression analysis of MAbs 1M5 and 1M25 by SDS-PAGE. The purified MAbs were resolved by reducing SDS-PAGE, followed by staining with Coomassie blue. M, protein molecular weight marker (kilodaltons); lane 1, MAb 1M25; lane 2, MAb 1M5.

The pattern diagram of MAb production using singe B cell antibody techniques is shown in Fig. 1A. Peripheral blood mononuclear cells (PBMCs) from SVA-inoculated and -immunized pigs were stained with fluorescein isothiocyanate (FITC)-labeled mouse anti-pig IgG and biotinylated SVA antigen followed by mouse antibiotin allophycocyanin (APC) antibodies, and SVA-specific (IgG^+^ SVA^+^) single B cells were sorted by flow cytometry (Fig. 1B). The V_H_ and V_L_ genes of the antibody were successfully amplified from the cDNA of sorted single B cells via nested PCR using the primers shown in Table 1, and the PCR products were then sequenced. The results showed that the V_H_ and V_L_ genes of 1M5 were 348 bp and 339 bp, respectively, and that the V_H_ and V_L_ genes of 1M25 were 354 bp and 348 bp, respectively. To produce a full-length porcine MAb, pairs of V_H_ and V_L_ genes were synthesized with codon optimization (for Cricetulus griseus) and cloned into corresponding expression vectors containing porcine IgG heavy- or light-chain constant region genes, respectively. The plasmids containing full-length porcine IgG heavy chain and light chain, respectively, were cotransfected into ExpiCHO-S cells for expression. To examine whether full-length porcine MAbs were successfully expressed and assembled, the expressed antibodies were purified from the supernatant and analyzed by sodium dodecyl sulfate-polyacrylamide gel electrophoresis (SDS-PAGE). As shown in Fig. 1C, the heavy and light chains of MAb 1M5 were approximately 52.8 kDa and 24.3 kDa, respectively, and the heavy and light chains of MAb 1M25 were approximately 53.3 kDa and 24.4 kDa, respectively, indicating that the produced porcine MAbs were indeed intact.

**TABLE 1:** Nested-PCR primers used for amplification of the V_H_ and V_L_ genes of pig IgG

Primer	Sequence (5′–3′)^*a*^
IgG V_H_ outer-F	GTTTCGGCTGAACTGGGTGGTC
IgG V_H_ outer-R	GGTCACTGRCTCGGGGAAGTAGC
IgG V_H_ inner-F	GGTGGAGTSTGGRGGAGGCCT
IgG V_H_ inner-R	CAGGGGGCCAGAGGGTAGACC
IgG V_L_ outer-F	ATGGCCTGGACGGTGCTTCTGATC
IgG V_L_ outer-R	CCTCCAGGTCACSGTCACG
IgG V_L_ inner-F	TCTCAGACTGTGATCCAGGAG
IgG V_L_ inner-R	GTCACTTATTAGACACACCAGGGTG

a R = A or G; S = C or G.

### Characterization of MAbs 1M5 and 1M25.

**FIG 2:**
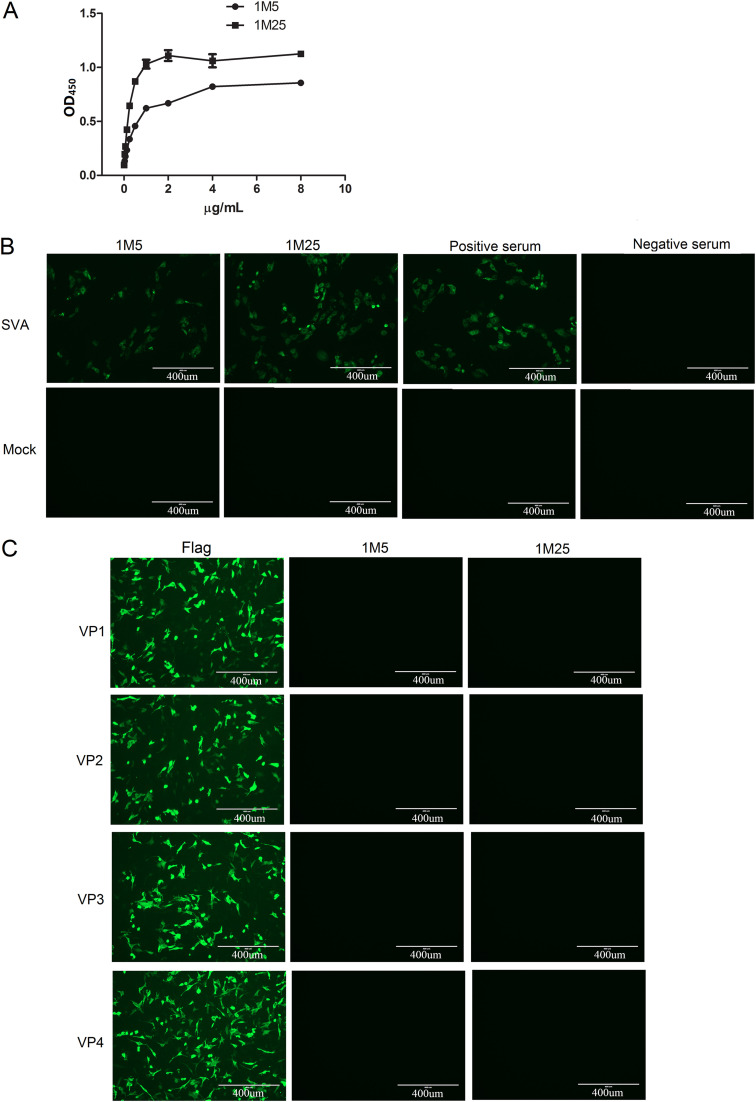
Characterization of the SVA-specific MAbs 1M5 and 1M25. (A) Detection of the reactivity of MAbs 1M5 and 1M25 to SVA antigen by indirect ELISA. The MAbs with different concentrations were added to SVA antigen-coated ELISA plates and incubated at 37°C for 1 h. Then, HRP-conjugated rabbit anti-pig antibody was added, and the OD_450_ values were measured using an automatic microplate reader. The results were obtained from three biological replicates (mean ± standard deviation). (B) Detection of the reactivity of MAbs 1M5 and 1M25 to SVA by IFA. BHK-21 cells were mock infected or infected with SVA and identified via IFA using 1M5, 1M25, anti-SVA-positive serum, and anti-SVA-negative serum. (C) Detection of the reactivity of MAbs 1M5 and 1M25 to SVA structural proteins by IFA. BHK-21 cells were transfected with plasmid containing the SVA VP1, VP2, VP3, or VP4 gene, respectively. The cells were then fixed and immunostained with 1M5, 1M25, and anti-Flag MAb followed by specific secondary antibody.

The reactivities of MAbs 1M5 and 1M25 to SVA antigen were determined using indirect ELISA. The results showed that both 1M5 and 1M25 (1 nM is equal to about 0.077 μg/mL) exhibited excellent affinity for SVA antigen, and the binding occurred in a dose-dependent manner (Fig. 2A). To further investigate whether MAbs 1M5 and 1M25 could identify SVA antigen in infected cells, IFA was performed. As shown in Fig. 2B, MAbs 1M5 and 1M25 could react with the SVA-infected cells and exhibit intense fluorescence similar to that of the SVA-positive serum, but no fluorescence was detected in the SVA-infected cells treated with SVA-negative serum or in the mock-infected cells, indicating that MAbs 1M5 and 1M25 could specifically recognize the viral antigen in SVA-infected baby hamster kidney 21 (BHK-21) cells. Additionally, Western blot analysis was also used to examine the reactivity of MAbs 1M5 and 1M25 to SVA, and the results showed that MAbs 1M5 and 1M25 did not react with denatured SVA capsid proteins and lysates of SVA-infected cells (data not shown). Together, these results indicate that MAbs 1M5 and 1M25 recognize the conformational epitopes of the viral capsid. To further determine the capsid protein recognized by MAbs 1M5 and 1M25, respectively, BHK-21 cells were separately transfected with plasmid containing VP1, VP2, VP3, or VP4, and then IFA was performed. The results (Fig. 2C) showed that strong immunofluorescence signals were observed in the transfected BHK-21 cells when stained with the anti-Flag antibody but not in the corresponding cells stained with MAb 1M5 or 1M25, indicating that MAbs 1M5 and 1M25 did not recognize individual capsid proteins. Taken together, these results demonstrate that MAbs 1M5 and 1M25 recognize the conformational epitopes that depend on the intact virion.

The neutralization activity of MAbs 1M5 and 1M25 against SVA was tested by VNT, and the result indicated that MAbs 1M5 and 1M25 could effectively neutralize SVA *in vitro* with a 50% inhibitory concentration (IC_50_) of 5.6 μg/mL and 0.75 μg/mL, respectively.

### Epitope specificity analysis of MAbs.

The epitope specificity of MAbs 1M5 and 1M25 was investigated by the ELISA additivity test. When the concentrations of MAbs are saturated for the antigen, the additivity index (AI) value will tend to be zero if the two MAbs bind to the same epitope but close to 100% if the two MAbs recognize different sites. The ELISA additivity test was carried out two times in triplicate in each plate, and the AI value was calculated from the mean of six values. ELISA additivity tests revealed that the AI value for MAbs 1M5 and 1M25 was 87 (Table 2), indicating that MAbs 1M5 and 1M25 recognize distinct epitopes.

**TABLE 2:** ELISA AI for 1M5 and 1M25 monoclonal antibodies

MAb	ELISA OD	Avg	AI^*a*^
1M5	0.362	0.364	
	0.313		
	0.335		
	0.378		
	0.401		
	0.393		
1M25	0.803	0.811	
	0.785		
	0.81		
	0.792		
	0.843		
	0.835		
1M5 + 1M25	1.12	1.099	87
	1.084		
	1.153		
	1.118		
	1.047		
	1.07		

a AI, additivity index.

### Determination of the cutoff, sensitivity, and specificity of the NAC-ELISA.

**FIG 3:**
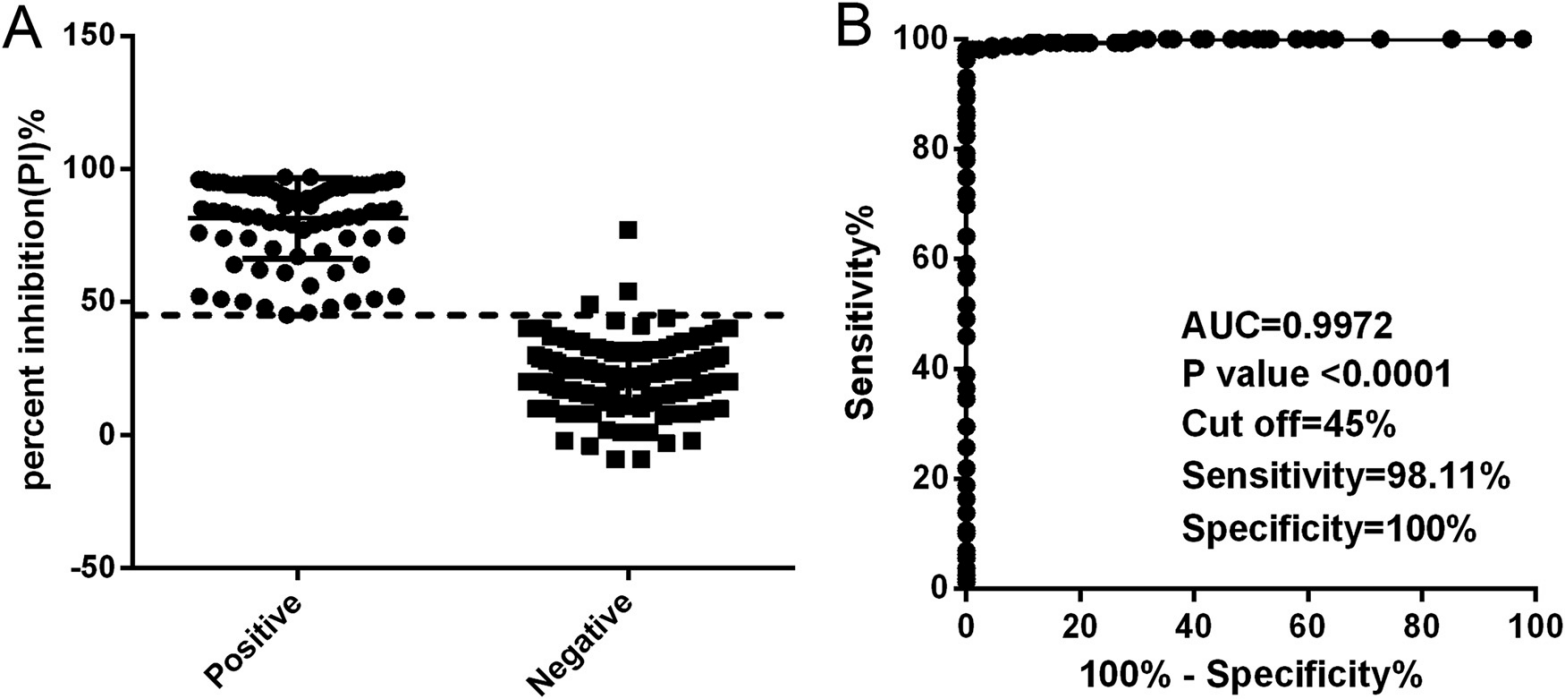
Receiver-operating characteristic (ROC) analysis for determination of the cutoff value of the NAC-ELISA. (A) Distribution of PI values for SVA antibody-negative and -positive serum samples. Each dot represents one serum sample. (B) ROC curve of PI values obtained by NAC-ELISA. The horizontal dotted line represents the optimal cutoff value with the best sensitivity and specificity. AUC represents area under the curve.

A panel of 159 negative sera and 88 positive sera was used to determine the cutoff value, sensitivity, and specificity of the NAC-ELISA. The percent inhibition (PI) values of individual animals are shown in Fig. 3A, and receiver-operating characteristic (ROC) curve analysis was carried out based on data from the NAC-ELISA results. The value of sensitivity plus specificity was maximal when the cutoff value for the NAC-ELISA was set as 45%, and the corresponding sensitivity and specificity were 98.11% and 100%, respectively (Fig. 3B). The area under the ROC curve was 0.9972 (95% confidence interval [CI] = 0.9931 to 1.000) with a *P* value of <0.0001. Therefore, the cutoff value of PI was set as 45%. Thus, serum samples with a PI of <45% were considered negative, and those with a PI of ≥45% were considered positive.

### Cross-reactivity and repeatability of NAC-ELISA.

**FIG 4:**
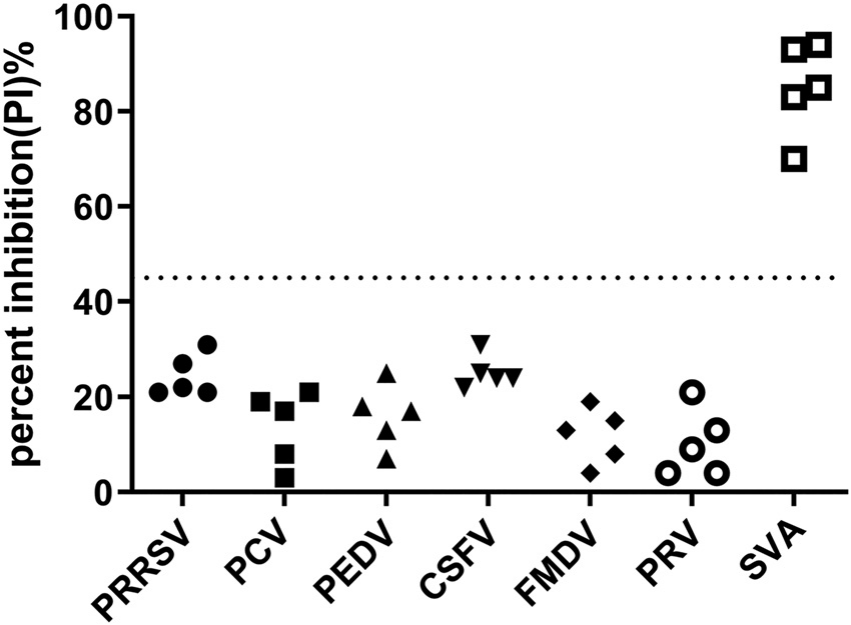
Cross-reactivity of the NAC-ELISA with seropositive samples against SVA and other swine viruses. Dispersion of individual PI values for PRRSV-positive sera (*n* = 5), PCV-positive sera (*n* = 5), PEDV-positive sera (*n* = 5), CSFV-positive sera (*n* = 5), FMDV-positive sera (*n* = 5), PRV-positive sera (*n* = 5), and SVA-positive sera (*n* = 5). The horizontal dotted line represents the cutoff value.

To evaluate the cross-reactivity of NAC-ELISA developed here, positive sera from PRRSV-infected swine (*n* = 5), porcine circovirus (PCV)-infected swine (*n* = 5), porcine epidemic diarrhea virus (PEDV)-infected swine (*n* = 5), CSFV-infected swine (*n* = 5), FMDV-infected swine (*n* = 5), and porcine pseudorabies virus (PRV)-infected swine (*n* = 5) were tested. The results demonstrated that all of the sera except the SVA-positive sera were negative with PI values lower than 45% (Fig. 4), indicating that the NAC-ELISA was specific for detecting SVA-specific antibodies and that there was no cross-reactivity with other virus-infected sera.

To evaluate the repeatability of NAC-ELISA developed here, the competitive ELISA reaction plates coated in the same and different batches were used, and eight serum samples were tested in triplicate. The inter- and intra-assay coefficients of variation (CV) for the NAC-ELISA were calculated. As shown in Table 3, the intrabatch CV% of eight serum samples ranged from 1.48% to 9.07% and the interbatch CV% of these samples ranged from 4.80% to 13.56%, suggesting that the NAC-ELISA has high reproducibility.

**TABLE 3:** Repeatability test of the NAC-ELISA

Sample ID^*c*^	Intra-assay value						Inter-assay value					
	1^*a*^	2	3	X^*b*^	SD	CV%^*d*^	1^*a*^	2	3	X^*b*^	SD	CV%^*d*^
1	30	25	29	28.0	2.16	7.72	25	28	20	24.3	3.30	13.56
2	38	41	35	38.0	2.45	6.45	35	33	38	35.3	2.05	5.82
3	18	20	16	18.0	1.63	9.07	15	20	18	17.7	2.05	11.63
4	76	81	82	79.7	2.62	3.29	73	79	82	78.0	3.74	4.80
5	84	86	83	84.3	1.25	1.48	86	75	82	81.0	4.55	5.61
6	68	74	71	71.0	2.45	3.45	63	74	73	70.0	4.97	7.10
7	54	55	60	56.3	2.62	4.66	59	63	52	58.0	4.55	7.84
8	87	91	84	87.3	2.87	3.28	92	85	82	86.3	4.19	4.85

a PI value.

b Mean value.

c ID, identifier.

d CV, coefficient of variation.

### Serum antibody dynamics in the experimentally infected animals.

**FIG 5:**
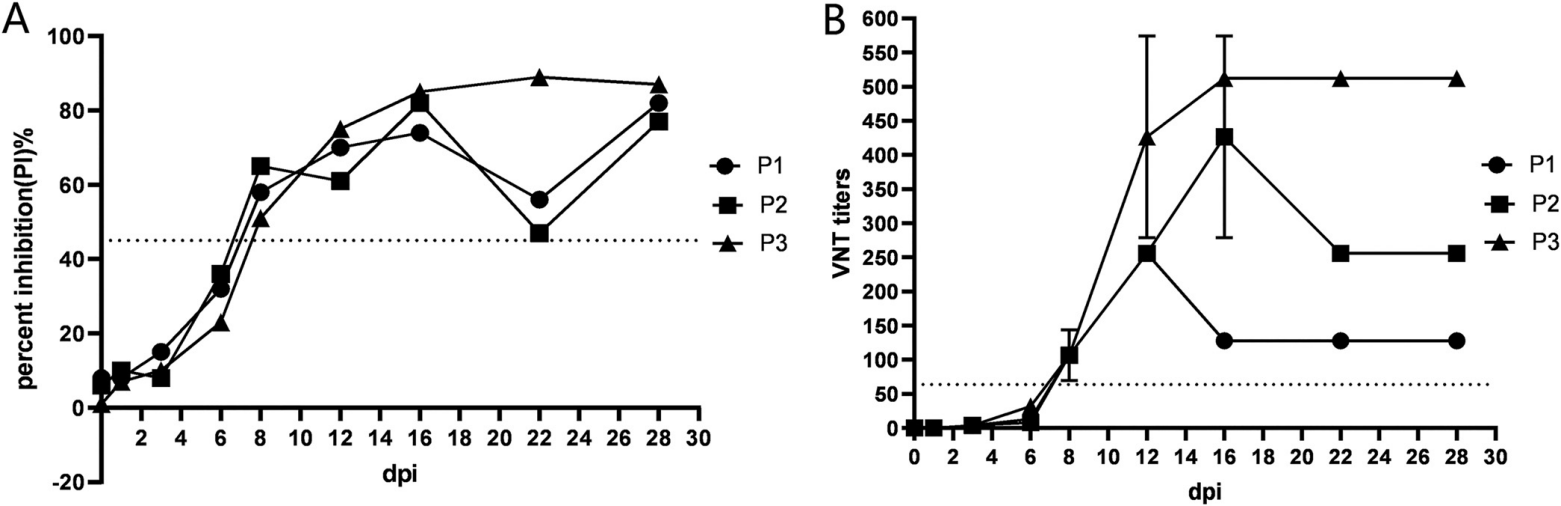
Detection of the SVA-specific antibody response for SVA-experimentally inoculated pigs by VNT and NAC-ELISA. (A) Sera from experimentally infected pigs were examined for the presence of antibodies to SVA by NAC-ELISA. The horizontal dotted line represents the cutoff value. (B) Sera from experimentally infected pigs were examined for the presence of neutralizing antibodies to SVA by VNT. The results were obtained from three biological replicates (mean ± standard deviation). The horizontal dotted line represents the cutoff value.

The sera from experimentally infected pigs were collected at different times postinfection and tested using the NAC-ELISA and the VNT. As shown in Fig. 5, the two methods exhibited similar dynamics of the SVA-specific immune response for the 3 tested pigs. All pigs showed a positive SVA-specific antibody response at 8 days postinfection (dpi) and remained positive until the end of the experiments (28 dpi) by NAC-ELISA and VNT methods. Comparison of the results of the NAC-ELISA with those of VNT indicated that the coincidence rate between the NAC-ELISA and VNT was 100% (27/27), demonstrating a 100% agreement between NAC-ELISA and VNT. Therefore, these results confirm that the NAC-ELISA is highly accurate.

### Comparison of diagnostic performances.

To verify the applicability of the NAC-ELISA, a total of 257 clinical swine serum samples collected from Gansu Province in 2021 were tested using the NAC-ELISA and VNT. As shown in Table 4, 191 sera were negative and 66 sera were positive by VNT, whereas 182 sera tested negative and 75 sera tested positive by the NAC-ELISA. Eleven sera were positive by the NAC-ELISA but were negative with VNT, and 2 sera were negative by the NAC-ELISA but were positive with VNT. The coincidence rate between the NAC-ELISA and VNT was approximately 95% (244/257). The strength of agreement between the NAC-ELISA and VNT was assessed by Cohen kappa coefficient, and the result demonstrated that there was good agreement between NAC-ELISA and VNT with a kappa value of 0.873.

**TABLE 4:** Comparison of the NAC-ELISA with VNT for the detection of SVA antibody in field serum samples

Test and result type	No. of samples with NAC-ELISA result		Total
	Negative	Positive	
VNT			
Negative	180	11	191
Positive	2	64	66
Total	182	75	257

## DISCUSSION

Although SVA has been silently circulating in pigs since 1988, it was not until 2007 that SVA was confirmed as the causative agent of vesicular disease in swine (1, 32). Since 2014, SVA has spread widely in many countries, resulting in vesicular lesions in swine and acute death in neonatal piglets (with a fatality rate of 30% to 70%) (12, 24). Therefore, the outbreak and epidemic of SVA seriously affected the healthy development of local pig industries and caused great economic losses to the pig industry (44). Moreover, previous studies showed that the historical SVA isolates failed to cause clinical symptoms in experimentally infected pigs (45, 46), but the contemporary SVA isolates can easily cause vesicular lesions in naturally or experimentally infected pigs (23, 46), suggesting that SVA is evolving toward a more virulent phenotype over time. Furthermore, the clinical symptoms of SVA infection are indistinguishable from those of FMD, which is an economically important and devastating animal disease. Thus, a rapid and specific diagnosis of SVA infection helps to rule out other exotic diseases and is necessary to its prevention and control. Here, we developed a sensitive and specific NAC-ELISA for the detection and diagnosis of SVA-specific antibodies based on two SVA-specific porcine MAbs.

Currently, a variety of serological detection methods for SVA have been reported, including VNT, IFA, indirect ELISA, and cELISA (27, 30–32, 47, 48), among which the indirect ELISA and cELISA are the most commonly used methods for the diagnosis of viral infection since these ELISA methods are simple, rapid, and easy to perform. However, these ELISAs are based on monoclonal or polyclonal antibodies derived from mouse or rabbit, which result in low specificity in view of the response to SVA differing in antigenic structure and preference from those of the natural host. Moreover, preparation of monoclonal and polyclonal antibodies is time-consuming and requires the slaughter of animals. To overcome these shortcomings, in this study, two recombinant porcine-derived MAbs against SVA, 1M5 and 1M25, were successfully produced and used to substitute for murine monoclonal antibody or rabbit polyclonal antibody to develop an ELISA method. These two porcine-derived MAbs were generated using a combination of SVA-specific single B cell sorting, RT-PCR amplification of IgG heavy- and light-chain genes of single B cells, and expression of antibodies in CHO-S cells. This method has many merits. First, the MAbs can be obtained from natural host animals without slaughter. Second, the MAbs isolated from single B cells can maintain the natively matched V_H_ and V_L_ pairing as it exists in animals. Third, the MAbs expressed in eukaryotic expression systems have many advantages including batch uniformity, native conformation, and economic large-scale production. Thus, MAbs 1M5 and 1M25 produced in this study were derived from swine with native conformation and can be mass produced to provide sufficient material for a variety of applications.

To evaluate the reactivity of MAbs 1M5 and 1M25 to SVA, indirect ELISA, IFA, and Western blot analysis were performed. Indirect ELISA results showed that MAbs 1M5 and 1M25 react with purified SVA antigen in a dose-dependent manner, and IFA and Western blotting results showed that MAbs 1M5 and 1M25 could specifically react with SVA antigen in SVA-infected BHK-21 cells in IFA but not in Western blotting, suggesting that MAbs 1M5 and 1M25 recognize the conformational epitopes of the viral capsid (Fig. 2). Moreover, to further determine the capsid protein recognized by MAbs 1M5 and 1M25, respectively, BHK-21 cells separately transfected with the VP1, VP2, VP3, or VP4 gene were tested by IFA. The results suggested that MAbs 1M5 and 1M25 did not recognize individual capsid protein, indicating that MAbs 1M5 and 1M25 recognize the conformational epitopes that depend on an intact virion (Fig. 2C). To detect whether MAbs 1M5 and 1M25 recognize different epitopes, an ELISA additivity test was carried out, and the results indicated that the binding of MAbs 1M5 and 1M25 was additive with an AI value of 87, suggesting that MAbs 1M5 and 1M25 recognize distinct epitopes (Table 2). However, further studies to identify the epitopes recognized by 1M5 and 1M25, respectively, were needed. Additionally, the neutralization activity of MAbs 1M5 and 1M25 was also tested by VNT, and the result showed that MAbs 1M5 and 1M25 could effectively neutralize SVA *in vitro*; in particular, MAb 1M25 exhibited ultrapotent neutralization with an IC_50_ of <1 μg/mL. Overall, the MAbs 1M5 and 1M25 obtained in this study were neutralizing MAbs that recognize intact virion-dependent conformational epitopes and can be used for ELISA, VNT, and IFA.

Since MAbs 1M5 and 1M25 bind to distinct epitopes, a NAC-ELISA for the detection of neutralizing antibodies against SVA was developed using MAb 1M5 as the capture antibody and MAb 1M25 as the detection antibody. The MAbs 1M5 and 1M25 were neutralizing MAbs and derived from the natural host, which gives the NAC-ELISA a good performance. Based on 159 negative sera and 88 positive sera, the sensitivity and specificity of the NAC-ELISA were estimated to be 98.11% and 100%, respectively, with a cutoff value of 45% PI (Fig. 3), values which were higher than those of a previously developed cELISA with a sensitivity and specificity of 96.9% and 98.2%, respectively (31). The possible reason may be that the cELISA was developed based on a murine MAb against SVA, while the NAC-ELISA described here was developed based on an SVA-specific porcine MAb which is closer to native conformation and has better reactivity to the antigen. However, considering that the number of known serum samples is limited, further studies are needed to accurately evaluate these parameters for the NAC-ELISA with more standard negative and positive serum samples. Additionally, serum samples from PCV-, PRRSV-, PEDV-, CSFV-, FMDV-, and PRV-infected swine had PI values lower than 45%, indicating that the NAC-ELISA was specific for detecting SVA-specific antibodies with no cross-reactivity to other virus-infected sera (Fig. 4). The inter- and intra-assay CV values for the NAC-ELISA were lower than 14%, demonstrating that the NAC-ELISA has good repeatability and reproducibility (Table 3). As far as we know, this is the first time that porcine-derived neutralizing MAbs have been reported to be used as the capture and detection antibodies in a competitive ELISA for detecting neutralizing antibodies against SVA.

To evaluate the performance of the NAC-ELISA, the sera collected at various days postinfection from pigs experimentally infected with SVA were tested using the NAC-ELISA, and the results were compared with the VNT results. All SVA-experimentally inoculated pigs displayed a positive SVA-specific antibody response at 8 dpi and remained positive until the end of the experiments by both methods (Fig. 5). The results were inconsistent with previous studies, in which the SVA-specific antibodies were first detected at 5 dpi (27, 47). The possible explanation for this discrepancy may be that the inoculation methods used in these studies were different. Joshi et al. reported that pigs inoculated with an SVA suspension (10^8.07^ 50% tissue culture infective doses [TCID_50_]/mL) via the oral (2 mL) and intranasal (1.5 mL to each nostril) routes can develop anti-SVA antibodies at 5 days postinoculation (47). Yang et al. reported that pigs inoculated with SVA-infected cell culture supernatant (1 × 10^8^ PFU/pig) or purified live SVA particles (5 × 10^11^ particles/pig) via coronary bands and intralingually exhibited a positive SVA-specific antibody response at 5 days postinoculation (27). However, in naturally infected pigs, less than 15% of animals showed SVA-specific antibody response (anti-SVA VP1) at 1 week after clinical signs, and over 70% of animals were seropositive at 3 weeks after clinical signs (32). Thus, the mode of SVA infection may influence the timing of the early antibody response. In this study, pigs were inoculated with SVA-infected cell culture supernatant (10^8.5^ TCID_50_/mL) via intramuscular injection behind the ear (2 mL to each ear). Although all pigs showed a positive SVA-specific antibody response at 8 to 28 dpi, the neutralizing antibody titers obtained by VNT in the experimentally inoculated pigs were low, especially in pig P1 (Fig. 5), which is inconsistent with the opinion that SVA infection is highly immunogenic and results in high antibody titers (15, 23, 47). However, the titers of neutralizing antibodies increased rapidly when the experimentally inoculated pigs were immunized with inactivated SVA at 28 dpi (data not shown), which is consistent with the view that high titers of anti-SVA neutralizing antibody in naturally infected pigs may be due to the circulation of SVA within the herd, leading to the constant viral exposure of the animals and consequently to immunological boosting (48). Additionally, previous studies have shown that VNT is sensitive and specific for detecting antibodies in SVA-infected pig serum (31, 48). For the diagnosis of SVA infection, the diagnostic sensitivity and specificity of the VNT were slightly higher than those of the murine monoclonal antibody-based cELISA (31). In the present study, comparison of the NAC-ELISA results with VNT showed that the coincidence rate between the NAC-ELISA and VNT was 100% (27/27), demonstrating a 100% agreement between NAC-ELISA and VNT. To further evaluate the performance of the NAC-ELISA, a panel of 257 clinical swine serum samples collected from Gansu Province in 2021 was tested using the NAC-ELISA, and the results were compared with the VNT results. The coincidence rate between the NAC-ELISA and VNT was approximately 95% (244/257), and the Cohen kappa coefficient between the NAC-ELISA and VNT was 0.873, indicating that there is a strong agreement between NAC-ELISA and VNT for clinical diagnostic performance. Thus, the NAC-ELISA developed in the current study is comparable to the VNT for SVA antibody detection and can replace VNT for large-scale screening since it is quicker, simpler, and less expensive to perform.

In conclusion, two porcine-derived MAbs against SVA were generated, and a competitive ELISA for the detection of neutralizing antibodies against SVA was successfully developed using these two porcine MAbs. The NAC-ELISA is SVA specific with no cross-reactivity to other related pathogens and has high sensitivity, specificity, and reproducibility for detecting SVA-specific antibody. Additionally, the results of NAC-ELISA were comparable to those obtained by VNT, suggesting that NAC-ELISA can be used as an alternative to VNT for detection of SVA antibodies. Therefore, the NAC-ELISA developed in this study may be of great value as a simple and reliable tool for serodiagnosis or surveillance of SVA and may facilitate the prevention and control of SVA.

## MATERIALS AND METHODS

### Serum samples.

A total of 159 serum samples from clinically healthy pigs were collected and tested to be negative for antibodies against SVA by the VNT (titer, <1:64). A total of 88 serum samples were collected from naturally infected or experimentally vaccinated pigs, which were tested and found to be positive for antibodies against SVA by VNT (titer, >1:64). These negative and positive serum samples were used to evaluate the sensitivity, specificity, and cutoff value of the NAC-ELISA. Twenty-seven serum samples obtained by serial blood sampling from pigs experimentally infected with SVA (3 pigs, 0 to 28 dpi) were used to compare the diagnostic performances of the NAC-ELISA and VNT. A total of 257 clinical swine serum samples collected from pig farms in Gansu Province in 2021 were used to assess the concordance between the NAC-ELISA and VNT. Additionally, six panels of serum samples (5 samples in each panel) from PRRSV-, PCV-, PEDV-, CSFV-, FMDV-, and PRV-infected swine were tested in this study.

### Preparation of SVA.

BHK-21 cells were infected with SVA strain HN/11/2017 at a multiplicity of infection (MOI) of 1, and virus was harvested at 48 h postinfection. Subsequently, the virus was inactivated with 3 mM binary ethylenimine (BEI; Sigma-Aldrich) at 30°C for 28 h, concentrated with 8% polyethylene glycol 6000 (PEG 6000; Sigma-Aldrich), and purified on 15 to 45% (wt/vol) sucrose density gradients. The virus band was collected and dialyzed in phosphate-buffered saline (PBS). The virus particles were quantified by measuring the absorbance at 260 nm and were divided into aliquots and stored at −80°C.

### Sorting of SVA-specific single B cells.

Heparinized blood was sampled from a pig with a high-level SVA-specific antibody response that had received SVA strain inoculation (HN/11/2017) and SVA vaccination (inactivated HN/11/2017) at 28-day intervals. The peripheral blood mononuclear cells (PBMCs) were isolated using 1.077 g/mL Histopaque (Sigma-Aldrich, USA) according to the manufacturer’s instructions. PBMCs were stained with biotinylated SVA antigen and FITC-labeled mouse anti-pig IgG (MyBioSource, USA) for 30 min at 4°C and subsequently with mouse antibiotin-APC (Miltenyi Biotec, San Diego, CA, USA) as the secondary antibody for a further 30 min at 4°C. The parallel staining of PBMCs lacking biotin-SVA antigen was used as fluorescence minus one (FMO) control. These stained PBMCs were sorted by flow cytometry (BD FACS Aria II; USA) using a 100-μm nozzle. SVA-specific single cells (SVA-APC positive and IgG-FITC positive) were sorted into 96-well PCR plates containing lysis buffer and then immediately transcribed into cDNA using SuperScript VILO MasterMix (Thermo Fisher Scientific, USA) according to the manufacturer’s instructions. The obtained cDNA samples were stored at −40°C for subsequent PCR amplification.

### Preparation of monoclonal antibodies.

The V_H_ and V_L_ genes of antibody were amplified separately by nested PCR using primers for porcine IgG (Table 1). The first round of PCR was carried out with 2.5 μL of cDNA as the template and the outer primer pairs. The second round of PCR was carried out with 5 μL of the first-round PCR product as the template and the inner primer pairs. The final PCR products were subsequently sequenced by Sanger sequencing. The V_H_ and V_L_ genes derived from single cells were synthesized (GenScript, Nanjing, China) with codon optimization (for *Cricetulus griseus*) and cloned into expression vectors pCH-pcDNA3.4 containing porcine IgG heavy chain constant region genes with His tag at the C terminus and pCL-pcDNA3.4 containing porcine lambda light-chain constant region genes, respectively, as described previously (42). The antibody-expressing plasmids for light chain and heavy chain were cotransfected into ExpiCHO-S cells using an ExpiFectamine CHO transfection kit (Invitrogen, USA) according to the manufacturer’s instructions. Cell culture supernatants were harvested at 10 days posttransfection, and antibodies were purified using a HisTrap Excel column (GE Life Sciences, USA) per the manufacturer’s instructions. The purified porcine MAbs were analyzed by SDS-PAGE, and the concentration of purified MAbs was measured at *A*_280_ by a NanoDrop 2000 spectrometer (Thermo Fisher Scientific, USA).

### Indirect ELISA.

The porcine-derived MAbs were examined for their ability to bind SVA antigen by indirect ELISA. Briefly, 96-well ELISA plates were coated with purified SVA antigen (0.2 μg/well) at 4°C overnight. The plates were washed three times with PBST (0.05% Tween 20 in PBS) and blocked with blocking buffer (1% bovine serum albumin [BSA] in PBST) at 37°C for 1 h. After washing three times with PBST, the porcine-derived MAbs at varied concentrations (0 to 8 μg/mL) were added and incubated at 37°C for 1 h. After washing three times with PBST, horseradish peroxidase (HRP)-conjugated rabbit anti-pig antibody (1:10,000; Bio-Rad) was added to each well and incubated at 37°C for 1 h. After washing three times with PBST, substrate 3,3′,5,5′-tetramethylbenzidine (TMB) was added, and color development was terminated after 15 min by the addition of 2 M H_2_SO_4_. The optical density was determined at 450 nm (OD_450_) on an automatic microplate reader (BioTek, Winooski, VT, USA).

### Indirect immunofluorescence assay.

BHK-21 cells seeded in 24 wells were infected with SVA HN/11/2017 at an MOI of 5 for 10 h or transfected with plasmid containing the VP1, VP2, VP3, or VP4 gene, respectively, using Lipofectamine 2000 for 24 h. The cells were fixed with 4% paraformaldehyde for 15 min, permeabilized with 0.5% Triton X-100 for 10 min, and then blocked with 5% BSA for 1 h. After washing with PBS, the cells were incubated with the indicated primary antibodies (porcine-derived MAbs at a concentration of 4 μg/mL or mouse anti-Flag MAb) at 37°C for 1 h. After washing with PBS, the cells were incubated with the fluorescence-conjugated secondary antibodies (FITC-conjugated goat anti-pig IgG [Sigma-Aldrich, USA] at a 1:1,000 dilution or FITC-conjugated goat anti-mouse IgG [Sigma-Aldrich, USA] at a 1:1,000 dilution) for 1 h at 37°C. After three washes with PBS, the cells were observed under the Evos FL imaging system (Life Technologies, USA).

### ELISA additivity test.

To test whether purified MAbs 1M5 and 1M25 recognize different epitopes, the ELISA additivity test was performed as previously described by Friguet et al. (49). The purified SVA antigen was applied as a coating onto a 96-well ELISA plate. MAbs 1M5 and 1M25 were added either separately or simultaneously, and the bound antibody was then detected by the addition of HRP-labeled goat anti-pig IgG (Abcam). The AI for a pair of MAbs was calculated according to the formula AI = {[2 × *A*_1+2_/(*A*_1_ + *A*_2_)] − l} × 100, where *A*_1_, *A*_2_, and *A*_1+2_ are the OD values of the first MAb alone, the second MAb alone, and the two MAbs together, respectively, in the ELISA. The AI value of ≥50% indicated that the two antibodies recognized different epitopes, while the AI value of <50% indicated that the two antibodies recognized the same epitope.

### Virus neutralizing test.

The neutralization activity of MAbs or sera against SVA was tested using a VNT on BHK-21 cells with SVA strain HN/11/2017. Briefly, purified antibodies were 2-fold serially diluted and incubated with 100 TCID_50_ of SVA in a 96-well cell culture plate for 1 h at 37°C. Then, 100 μL of BHK-21 cells (5 × 10^4^ cells) in Dulbecco’s modified Eagle’s medium (DMEM) were added to each well, and the plates were incubated at 37°C for 72 h in a 5% CO_2_ incubator. The cells were fixed with cold acetone-methanol (volume ratio = 1:1) and stained with a 0.2% crystal violet solution. The neutralization titer for MAb was expressed as 50% inhibitory concentration (IC_50_) and calculated as the final antibody concentration to neutralize 100 TCID_50_ of SVA in 50% of the wells. An IC_50_ value of ≥50 μg/mL was regarded as nonneutralization activity. The neutralizing activity of sera was detected using an endpoint dilution assay. The neutralizing antibody titer was determined as the highest serum dilution that neutralized 100 TCID_50_ of SVA in 50% of the wells. Sera with a neutralizing antibody titer of ≥64 were regarded as positive.

### Biotinylation of MAb 1M25.

Purified MAb 1M25 was tagged with biotin using EZ-Link Sulfo-NHS-LC-biotin reagent (Thermo Fisher Scientific, USA) per the manufacturer’s protocols, and the biotinylated MAb 1M25 was named Bio-1M25.

### Development of NAC-ELISA.

A NAC-ELISA for SVA-specific neutralizing antibody detection was developed based on the newly generated MAbs 1M5 and 1M25. The optimal concentrations of the capture antibody (1M5), SVA antigen, detection antibody (Bio-1M25), and HRP-labeled streptavidin were determined by checkerboard titration, and the serum dilution, the incubation time, and the blocking buffer were optimized based on the ratio of the absorbance reading of negative and positive sera. The NAC-ELISA was performed under optimal conditions. Briefly, 96-well ELISA plates were coated with 2 μg/mL of 1M5 diluted in carbonate-bicarbonate buffer and incubated overnight at 4°C. After washing three times with PBST, 100 μL (0.5 μg/mL) purified SVA antigen diluted in PBS was added to each well and the plates were incubated for 2 h at room temperature. After washing three times with PBST, the plates were blocked with blocking buffer (1% bovine serum albumin [BSA] and 5% sucrose in PBST) at 37°C for 1 h. After washing three times with PBST, 1:10-diluted sera (the known positive sera, negative sera, and test sera) in PBST and the blank control (PBST) were added (50 μL/well) to the ELISA plates, and then 50 μL/well of Bio-1M25 (0.043 μg/mL) was added. The plates were incubated at 37°C for 30 min with agitation and washed five times with PBST. HRP-conjugated streptavidin (1:30,000; GenScript, Inc.) was added to the wells and incubated at 37°C for 15 min. After washing five times with PBST, TMB substrate was added and incubated at 37°C for 15 min and the reaction was terminated by the addition of 2 M H_2_SO_4_. The OD value was measured at 450 nm (OD_450_) using an automatic microplate reader (BioTek). The results were expressed as percent inhibition (PI), calculated by the following formula: PI (%) = [1 − (absorbance value of test sample/absorbance value of blank control)] × 100%.

### Statistical analysis.

ROC curve analysis was carried out to determine the cutoff value, sensitivity, and specificity of the NAC-ELISA by using 159 sera from uninfected animals and 88 sera from naturally infected or experimentally vaccinated animals.

The Cohen kappa coefficient was used to determine the strength of agreement between NAC-ELISA and VNT.

Statistical analysis and data plotting were performed using GraphPad Prism software (version 6.0; GraphPad Software, Inc., La Jolla, CA). Cohen kappa coefficient analysis was performed using SPSS v19.0 software (IBM SPSS Inc., Chicago, IL).

### Ethics statement.

All animal experiments were performed according to the management guidelines of the Gansu Ethical Review Committee (license no. SYXK-GAN-2014-003), and this study was approved by the Animal Ethics Committee of LVRI, Chinese Academy of Agricultural Sciences (permit no. LVRIAEC2020-008).

### Data availability.

The sequences of MAb 1M5 and 1M25 are available from the corresponding author upon reasonable request.
